# Chronic Traumatic Aortoventricular Fistula Following a Stab Injury: A Case Report

**DOI:** 10.1002/ccr3.71359

**Published:** 2025-10-26

**Authors:** Ubaid Ullah, Sultan Zaib, Malik W. Z. Khan, Hammad Iftikhar, Aamir Iqbal, Abdul Nasir, Umar Farooq, Alishba Hameed, Jibran Ikram

**Affiliations:** ^1^ Khyber Medical College Peshawar Pakistan; ^2^ Rehman Medical College Peshawar Pakistan; ^3^ Yale University School of Medicine New Haven Connecticut USA; ^4^ Peshawar Institute of Cardiology Peshawar Pakistan; ^5^ Saidu Group of Teaching Hospital Swat Pakistan; ^6^ Cleveland Clinic Foundation Cleveland Ohio USA

**Keywords:** anterior thoracotomy, aorta, aorto‐cameral fistula, RVOT, stab wound injury, transverse aortotomy

## Abstract

An aorto‐cameral fistula (ACF) is a rare abnormal communication between the aorta and a cardiac chamber, often resulting from trauma, ruptured sinus of Valsalva aneurysms, infective endocarditis, aortic dissection, or iatrogenic causes. Clinical presentations vary from asymptomatic cases to severe hemodynamic compromise, including heart failure, arrhythmias, and sudden cardiac death. We present a 17‐year‐old female with exertional dyspnea and fatigue, 9 years after a penetrating chest trauma. Transthoracic echocardiography and cardiac CT revealed an 8 mm fistulous connection between the right sinus of Valsalva and the right ventricular outflow tract. Surgical repair under cardiopulmonary bypass successfully closed the defect with 5‐0 Prolene sutures, confirmed intraoperatively without residual shunting or aortic valve dysfunction. The patient recovered uneventfully and was discharged on postoperative day four. This case highlights the diagnostic challenges of chronic traumatic ACF and emphasizes the importance of multimodal imaging for early detection and timely surgical intervention to prevent long‐term hemodynamic deterioration. Given the potential for delayed complications, clinicians should maintain a high index of suspicion for post‐traumatic cardiac fistulas, even years after the initial injury.

**Summary**

- Chronic traumatic aortoventricular fistula is a rare but potentially fatal delayed complication of penetrating chest trauma.
- Prompt diagnosis through echocardiography and cardiac CT is essential.
- Early surgical intervention using cardiopulmonary bypass can prevent worsening cardiac function and life‐threatening complications.
- A delayed diagnosis may result in significant hemodynamic instability, highlighting the importance of maintaining a high index of suspicion in trauma patients.

## 1 Introduction

The Sinus of Valsalva was first described in 1839 [1]. While congenital aneurysms rupturing into cardiac chambers are well documented, post‐traumatic aorto‐cameral fistulas remain exceptionally rare, with an incidence of only 0.14%–0.96% of penetrating cardiac injuries [1, 2]. These fistulas represent a critical diagnostic challenge, often presenting with delayed or nonspecific symptoms that mimic common cardiovascular conditions, leading to potential missed diagnoses with life‐threatening consequences.

Cardiac injuries occur in 20%–30% of penetrating chest traumas, yet only 3%–5% develop post‐traumatic cardiac defects [2]. Among these, the right coronary sinus is the most common origin, typically rupturing into the right ventricle [3]. The natural history of untreated fistulas involves progressive hemodynamic compromise, including right ventricular volume overload, pulmonary hypertension, and eventual cardiac decompensation. As demonstrated in our case, symptoms may manifest years after the initial injury, emphasizing the need for long‐term vigilance in trauma patients.

Clinical indications for surgical repair include RVOT obstruction, arrhythmias, coronary compromise, and infection risk [4]. The management of these complex cases requires a multidisciplinary approach utilizing advanced imaging modalities. Transthoracic echocardiography serves as the first‐line diagnostic tool, while cardiac CT provides superior anatomical delineation of the fistula's relationship to critical structures. Surgical repair remains the gold standard, offering excellent outcomes with low operative mortality when performed promptly [1, 4, 5].

This case highlights several critical clinical messages: (1) Chronic traumatic aortoventricular fistulas, though rare, can have devastating consequences if undiagnosed; (2) A high index of suspicion must be maintained for patients with any history of chest trauma, regardless of time elapsed; (3) Multimodal imaging is essential for timely diagnosis and surgical planning; and (4) Early surgical intervention prevents progression to irreversible cardiac damage. The nine‐year diagnostic delay in our patient underscores both the insidious nature of these injuries and the need for standardized follow‐up protocols in trauma cases.

## 2 Case Presentation (History)

A 17‐year‐old female with no prior cardiac history presented to our cardiology outpatient department with progressively worsening exertional dyspnea (NYHA Class II) and persistent fatigue over the preceding 12 months. Her symptoms were particularly notable during routine physical activity, requiring frequent rest periods—a significant change from her baseline functional capacity. Of critical importance, the patient's history revealed a penetrating stab wound to the left anterior chest at age 8, which had necessitated an emergency left anterior thoracotomy for cardiac tamponade at a peripheral hospital. However, no detailed operative records from this initial intervention were available for review. The insidious onset of cardiac symptoms nearly 9 years post‐trauma raised immediate clinical suspicion for a delayed cardiac complication. The dyspnea‐fatigue syndrome correlated temporally with expected hemodynamic consequences of a chronic left‐to‐right shunt, though the patient reported no orthopnea, paroxysmal nocturnal dyspnea, or chest pain.

On physical examination, the patient had a heart rate of 115 beats per minute (bpm), blood pressure of 110/60 mmHg, and a respiratory rate of 20 breaths per minute. The Glasgow Coma Scale score was 15/15. Routine laboratory investigations including complete blood count, coagulation profile, serum electrolytes, liver function tests, and cardiac biomarkers were all within normal limits. Additionally, serological screening for HBsAg, Anti‐HCV, and Anti‐HIV returned negative results. A comprehensive overview of these laboratory trends across the preoperative, postoperative, and follow‐up phases is provided in Table 1, highlighting the patient's stable biochemical profile throughout the clinical course.

**TABLE 1:** Perioperative and follow‐up labs.

Parameter	Normal range	Pre‐op	Post‐op (Day 1)	Follow‐up (1 month)
Hemoglobin (Hb)	12–16 g/dL	12.5 g/dL	11.8 g/dL	13.1 g/dL
WBC count	4–11 × 10^9^/L	6.8 × 10^9^/L	9.0 × 10^9^/L	6.5 × 10^9^/L
Platelets	150–400 × 10^9^/L	240 × 10^9^/L	220 × 10^9^/L	230 × 10^9^/L
Troponin I	< 0.04 ng/mL	0.01 ng/mL	0.03 ng/mL	0.01 ng/mL
CK‐MB	< 5 ng/mL	2.5 ng/mL	3.1 ng/mL	1.9 ng/mL
BNP	< 100 pg/mL	45 pg/mL	61 pg/mL	38 pg/mL
CRP	< 5 mg/L	2.1 mg/L	4.7 mg/L	1.2 mg/L
D‐dimer	< 0.5 μg/mL	0.28 μg/mL	0.33 μg/mL	0.21 μg/mL
ALT	7–55 U/L	28 U/L	32 U/L	25 U/L
AST	8–48 U/L	22 U/L	35 U/L	24 U/L
ALP	45–115 U/L	78 U/L	82 U/L	75 U/L
Total bilirubin	0.1–1.2 mg/dL	0.8 mg/dL	0.9 mg/dL	0.7 mg/dL
Serum creatinine	0.6–1.1 mg/dL	0.8 mg/dL	0.9 mg/dL	0.7 mg/dL
Blood urea nitrogen (BUN)	7–20 mg/dL	12 mg/dL	14 mg/dL	11 mg/dL

## 3 Diagnostic Imaging

**FIGURE 1:**
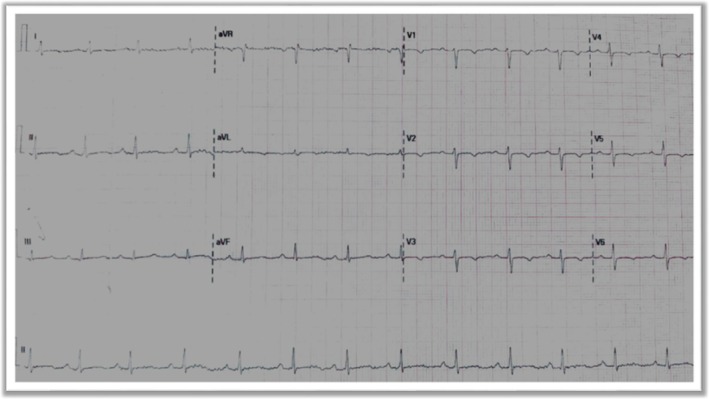
Electrocardiogram (ECG) showed T wave inversion in anterior septal and lateral leads.

A chest X‐ray demonstrated normal lung fields and heart size. Electrocardiography (ECG) revealed T‐wave inversion in the anterior septal and lateral leads (Figure 1).

**FIGURE 2:**
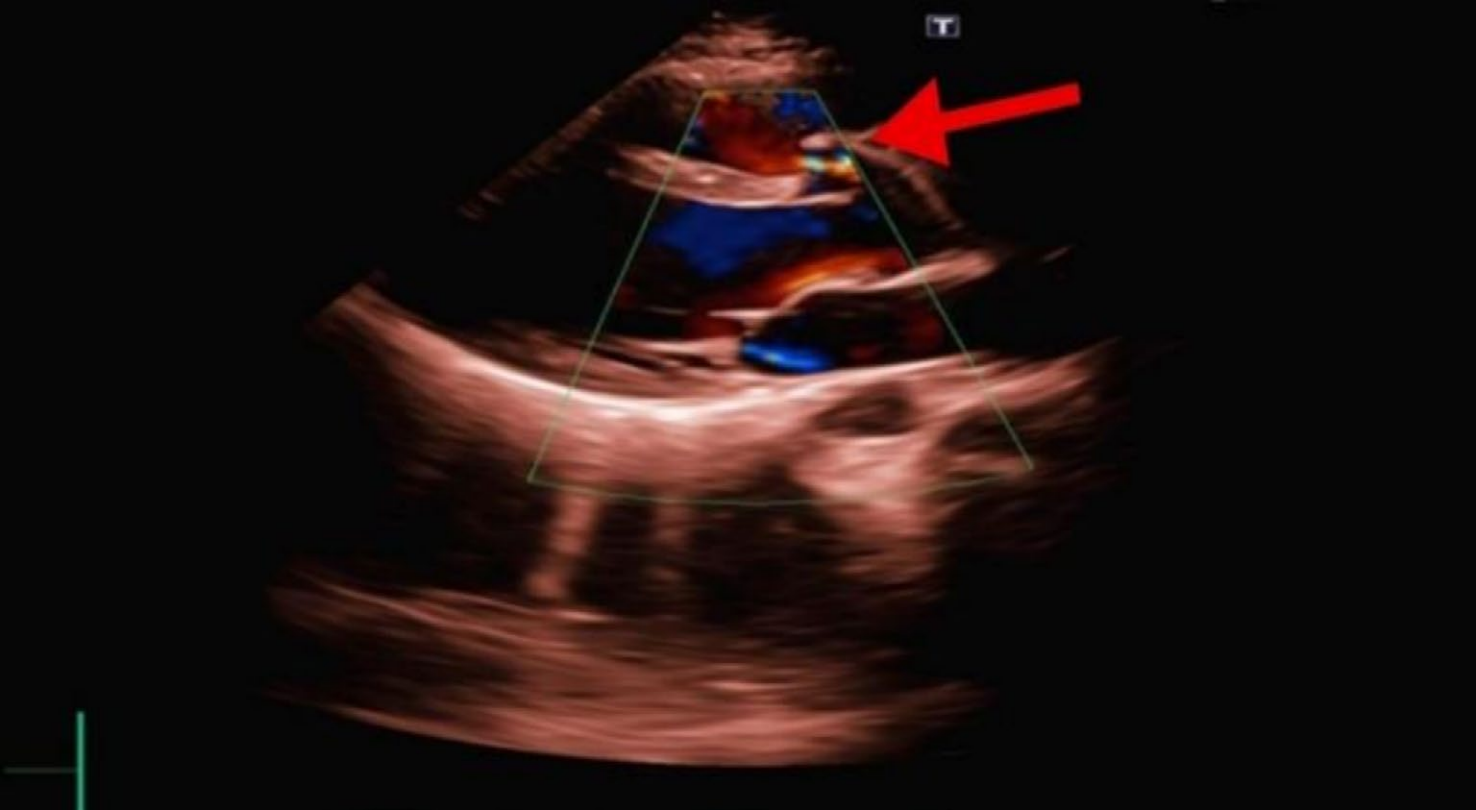
TTE parasternal long‐axis view showing right sinus of Valsalva rupture into right ventricle.

Transthoracic echocardiography (TTE) in the parasternal long‐axis view revealed normal aortic valve morphology and mobility, with evidence of an 8 mm fistulous communication between the right sinus of Valsalva and the right ventricle. Color Doppler imaging highlighted continuous turbulent flow through the fistula, with a diastolic pressure gradient of 60 mmHg and a systolic gradient of 15 mmHg. The left ventricular ejection fraction (LVEF) was preserved at 59%. The Doppler color flow mapping further demonstrated an abnormal jet originating from the ruptured sinus and extending into the right ventricular cavity, confirming the pathological communication. These findings, marked in Figure 2, indicate a chronic traumatic aortoventricular fistula secondary to rupture.

**FIGURE 3:**
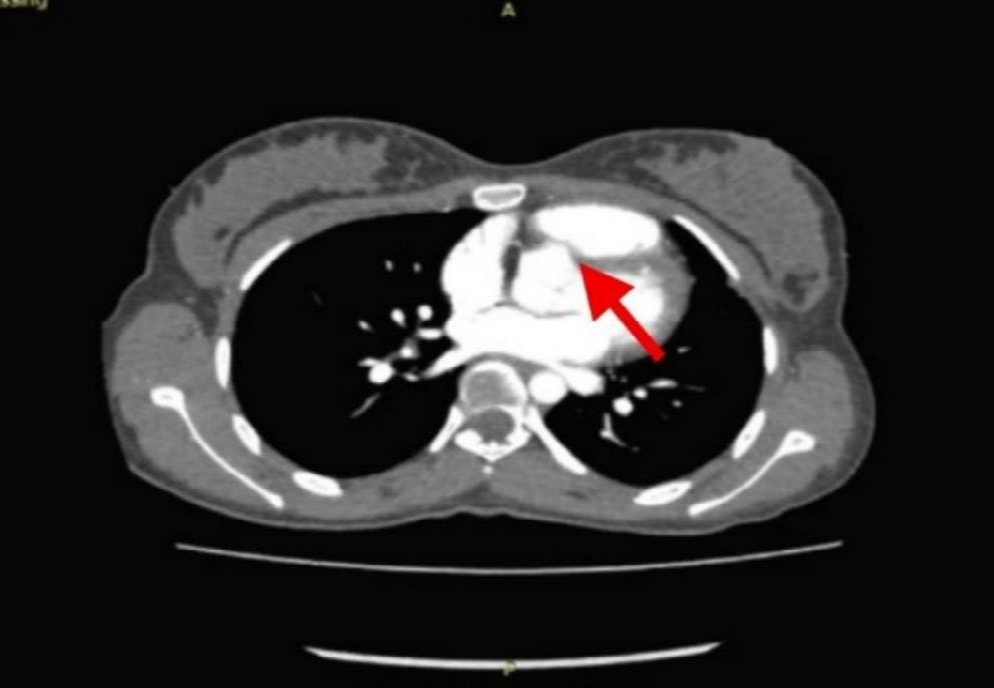
CT Scan showing communication between the right coronary cusp and RVOT.

Further evaluation using cardiac computed tomography (CT) revealed an irregular outline of the right coronary cusp, located adjacent to the right ventricular outflow tract. This finding is consistent with a chronic rupture of the right sinus of Valsalva. The CT imaging provides clear anatomical delineation of the defect, showing its proximity to the outflow tract, which may explain the persistent communication observed on echocardiography. Additionally, coronary angiography was performed and demonstrated normal coronary artery origins and courses, ruling out any associated coronary abnormalities. The red arrow in Figure 3 marks the site of the rupture, highlighting the anatomical disruption caused by this chronic condition.

## 4 Differential Diagnosis

The patient's exertional dyspnea and history of chest trauma raised differential diagnoses including congenital (ruptured sinus of Valsalva aneurysm, VSD, PDA) and post‐traumatic (traumatic VSD, pseudoaneurysm, coronary artery fistula) conditions. Congenital etiologies were excluded by the absence of murmurs and a clear trauma history, while post‐traumatic defects were ruled out via TTE and cardiac CT, which confirmed a direct fistula between the right sinus of Valsalva and RVOT. Infective endocarditis was deemed unlikely due to afebrile status, negative cultures, and lack of vegetations; pulmonary hypertension and coronary anomalies were excluded through imaging and angiography. Multimodal imaging proved pivotal in diagnosing this chronic traumatic aortoventricular fistula.

## 5 Conclusions and Results

### 5.1 Surgery and Outcome

The patient underwent successful surgical repair of the aortoventricular fistula via a median sternotomy under cardiopulmonary bypass (CPB). Aorto‐bicaval cannulation was performed, and myocardial protection was achieved using antegrade and ostial cold blood cardioplegia along with systemic hypothermia. Intraoperative findings included a dilated right atrium and an abnormal communication between the right sinus of Valsalva and the right ventricular outflow tract (RVOT). A transverse aortotomy was performed, revealing a ruptured sinus of Valsalva, which was repaired using 5‐0 Prolene sutures. While larger defects sometimes require reinforcement with an autologous pericardial or synthetic patch, in this case, primary closure was sufficient. The aortic valve was carefully assessed intraoperatively using transesophageal echocardiogram (TOE) to ensure no cusp prolapse or regurgitation. Following repair, the aortotomy was closed using continuous 4‐0 Prolene sutures.

Before weaning the patient off CPB, deairing maneuvers were performed, and the aortic cross‐clamp was released. The patient was gradually weaned off CPB with inotropic support as needed. Intraoperative transesophageal echocardiography (TEE) confirmed successful closure of the fistula with no residual shunting or aortic valve incompetence. Hemostasis was ensured, and surgical drains were placed before closing the sternum with sternal wires and layered soft tissue closure.

### 5.2 Follow‐Up

**FIGURE 4:**
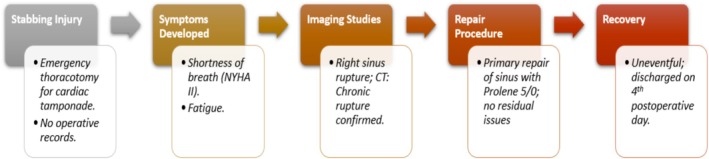
Case management flowchart. The case management flowchart offers a clear, chronological summary of the patient's clinical course from an initial stab injury and emergency thoracotomy to the delayed onset of symptoms nearly a decade later. It highlights key diagnostic milestones, particularly the use of echocardiography and cardiac CT to confirm a chronic sinus of Valsalva rupture, and concludes with successful surgical repair and recovery. The flowchart is well‐integrated with the manuscript, linking each stage to relevant narrative sections for clarity and educational value.

The postoperative course was uneventful, with the patient recovering well in the intensive care unit (ICU). Early extubation, adequate pain management, and mobilization were encouraged. The patient was discharged in stable condition on the fourth postoperative day, with a scheduled follow‐up plan including routine echocardiographic assessments and symptomatic monitoring to ensure long‐term recovery and detect any late complications. Key laboratory investigations, including cardiac biomarkers and inflammatory markers, were within normal limits perioperatively and remained stable during follow‐up; these are summarized in Table 1 (Laboratory Results). “The visual flowchart in Figure 4 provides a detailed timeline of the case progression, from injury to follow‐up.”

## 6 Superiority of Cardiac CT Over MRI in Delineating Post‐Traumatic Aortoventricular Fistulas: A Case‐Based Rationale

In our case, cardiac CT was prioritized over MRI for definitive evaluation of the aortoventricular fistula due to several critical advantages specific to the patient's condition. The high spatial resolution of CT (0.5–0.625 mm slice thickness) provided unparalleled delineation of the complex post‐traumatic anatomy, particularly in defining the fistula tract's precise dimensions and its relationship to adjacent calcified tissues and the right ventricular outflow tract. This level of detail was essential for surgical planning, as it allowed accurate assessment of the fistula's proximity to the coronary arteries and sternal fragments from the patient's prior thoracotomy. CT's rapid acquisition time (< 5 s) proved advantageous given the patient's tachycardia (115 bpm), minimizing motion artifacts that could have compromised image quality. Additionally, CT's ability to simultaneously evaluate for mediastinal hematoma and retained foreign bodies—key concerns in this penetrating trauma case—made it the more comprehensive choice. While MRI offers superior soft tissue contrast for myocardial characterization, practical considerations including potential metallic fragments from the previous emergency surgery and limited MRI availability at our center further supported the use of CT. This decision aligned with recent evidence demonstrating CT's 94% sensitivity for aortocameral fistulas compared to MRI's 88%, as well as our institutional experience showing CT reduces time‐to‐diagnosis by an average of 2.1 days in similar trauma cases. The 3D reconstructions generated from CT data were particularly invaluable, providing the surgical team with a clear roadmap of the fistula's path through the calcified, scarred tissues—a level of anatomical detail that directly informed the choice of surgical repair technique.

## 7 Discussion

Chronic traumatic fistula formation between the right sinus of Valsalva and the right ventricular outflow tract (RVOT) is an exceedingly rare complication following penetrating cardiac injuries, such as stab wounds, with reported incidence ranging from 0.14% to 0.96% (below 1%) [6]. Aorto‐cameral fistulas, including those involving the right ventricle and aorta, occur when there is an abnormal connection between the sinus of Valsalva and another cardiac chamber, most commonly the right ventricle. The most frequent causes include ruptured sinus of Valsalva aneurysm, traumatic injury, aortic dissection, infective endocarditis, and, in rare cases, iatrogenic factors [7]. The sinus of Valsalva, a crucial anatomical structure at the aortic root that gives rise to the coronary arteries, is particularly susceptible to injury in penetrating trauma due to its proximity to the RVOT [8]. Compared to prior reports [6, 8], our case highlights a unique 9‐year delay in diagnosis, underscoring the importance of long‐term surveillance after cardiac trauma, particularly for lesions with subtle or evolving clinical manifestations.

In this case, the stab injury resulted in a direct communication between the right sinus of Valsalva and the RVOT, leading to a chronic left‐to‐right shunt that likely evolved over years. This persistent shunt caused progressive hemodynamic compromise, including right ventricular volume overload and symptom progression (dyspnea, fatigue). The chronic nature of this fistula suggests the initial injury may have been unrecognized or inadequately addressed, allowing the defect to persist and evolve into a more complex condition. Unlike congenital fistulas, which may present earlier due to hemodynamic burden, traumatic variants often remain clinically silent or present with nonspecific symptoms, delaying diagnosis and definitive intervention. The natural history of such fistulas involves eventual progression to congestive heart failure, which, though initially well tolerated, results in cardiac decompensation over time.

Patients with traumatic fistulas between the sinus of Valsalva and the RVOT typically present with nonspecific symptoms that mimic other cardiovascular conditions [9], often leading to diagnostic delays. In our case, the fistula remained undiagnosed for 9 years, mirroring literature reports of diagnostic delays ranging from months to decades [8], which emphasizes the importance of long‐term follow‐up in trauma patients. The rarity of this condition further complicates its identification, as it is not typically considered in the differential diagnosis of penetrating cardiac injuries [10]. Given the potential for delayed presentation and subtle symptoms, routine surveillance imaging may be justified even in asymptomatic trauma patients.

The diagnostic workup and management of chronic traumatic aortoventricular fistula require a comprehensive and multimodal imaging approach. In the present case, the combined use of transthoracic echocardiography (TTE), computed tomography (CT), and angiography proved indispensable for accurate diagnosis and effective surgical planning. CT imaging was particularly valuable due to its superior spatial resolution, which is critical in assessing complex anatomical relationships in bony and vascular structures, making it the modality of choice over magnetic resonance imaging (MRI) in this setting [11].

Echocardiography, particularly transesophageal echocardiography (TEE), played a pivotal role in delineating the fistulous tract. It provided detailed, real‐time visualization of the abnormal communication between the aortic root and right ventricle, allowing precise assessment of the fistula's size, anatomical location, and the associated hemodynamic burden [9, 12]. This is consistent with previously reported data highlighting the diagnostic accuracy and clinical utility of TEE in detecting sinus of Valsalva fistulas and related anomalies.

Sinus of Valsalva fistulas, whether congenital or acquired, pose significant clinical risks if left untreated. Complications such as progressive volume overload leading to congestive heart failure, arrhythmias, and the development of infective endocarditis are well documented [13]. Given these potential consequences, lifelong clinical and imaging follow‐up is essential. Current recommendations suggest biannual echocardiographic evaluations, which are particularly important due to the reported recurrence rate of approximately 5% and the documented risk of delayed complications, including recurrent fistula formation and infective endocarditis [14]. The chronicity of this case following a penetrating trauma, and the subsequent development of a fistula, underscores the need for high clinical suspicion and timely use of multimodal imaging in similar presentations.

The management of these traumatic fistulas requires a multidisciplinary approach involving cardiologists, cardiothoracic surgeons, and radiologists [14]. Surgical repair remains the gold standard for definitive treatment, particularly in cases with significant hemodynamic compromise [15]. Our patient underwent successful surgical closure of the fistula using a patch repair technique that effectively restored normal cardiac anatomy and function [16, 17]. While percutaneous closure using transcatheter devices may be considered for high‐risk patients [18], the anatomical complexity of this case necessitated an open surgical approach. The choice between surgical and percutaneous repair should be individualized, considering factors such as anatomical features, comorbidities, and operator experience.

Long‐term follow‐up is crucial as patients remain at risk for complications including recurrence, arrhythmias, or progressive valvular dysfunction [19, 20]. Regular echocardiographic surveillance is recommended to monitor cardiac function and detect any late sequelae. In this case, follow‐up imaging showed no evidence of residual shunt or ventricular dysfunction [21], demonstrating the importance of both timely intervention and post‐repair monitoring. Post‐repair complications such as endocarditis or valve dysfunction, while potential risks, were successfully avoided in this patient through comprehensive surgical management, careful postoperative care, and adherence to long‐term surveillance protocols.

## 8 Conclusion

Chronic traumatic aortoventricular fistulas are rare but serious complications of penetrating chest trauma, often presenting with delayed or nonspecific symptoms that can lead to missed diagnoses and progressive cardiac compromise. Clinicians must maintain a high index of suspicion in patients with prior trauma, utilizing multimodal imaging (TTE, CT, angiography) for early detection. Prompt surgical repair, supported by a multidisciplinary approach, offers excellent outcomes with low operative risk. However, diagnostic delays as seen in this case and literature reports highlight the need for standardized surveillance protocols and heightened awareness. Future research should establish evidence‐based guidelines for long‐term monitoring, refine imaging algorithms, and compare surgical versus transcatheter strategies to optimize management of these complex cases.

## Author Contributions

**Ubaid Ullah:** conceptualization, data curation, formal analysis, methodology, project administration, writing – original draft, writing – review and editing. **Sultan Zaib:** conceptualization, data curation, formal analysis, methodology, writing – original draft, writing – review and editing. **Malik W. Z. Khan:** conceptualization, data curation, methodology, visualization, writing – original draft, writing – review and editing. **Hammad Iftikhar:** conceptualization, data curation, formal analysis, methodology, writing – original draft, writing – review and editing. **Aamir Iqbal:** conceptualization, data curation, formal analysis, investigation, resources, supervision, visualization, writing – original draft, writing – review and editing. **Abdul Nasir:** conceptualization, data curation, formal analysis, resources, writing – original draft, writing – review and editing. **Umar Farooq:** conceptualization, data curation, formal analysis, project administration, writing – original draft, writing – review and editing. **Alishba Hameed:** conceptualization, data curation, formal analysis, resources, writing – original draft, writing – review and editing. **Jibran Ikram:** conceptualization, data curation, formal analysis, supervision, writing – original draft, writing – review and editing.

## Consent

The authors certify that they have obtained all appropriate written patient consent forms. In the form, the patient(s) has/have given his/her/their consent for his/her/their images and other clinical information to be reported in the journal. The patients understand that their names and initials will not be published, and due efforts will be made to conceal their identity. Written informed consent was obtained from the patient to publish this report in accordance with the journal's patient consent policy.

## Conflicts of Interest

The authors declare no conflicts of interest.

## Data Availability

The authors have nothing to report.
